# Cyanide Production by *Chromobacterium piscinae* Shields It from *Bdellovibrio bacteriovorus* HD100 Predation

**DOI:** 10.1128/mBio.01370-17

**Published:** 2017-12-19

**Authors:** Wonsik Mun, Heeun Kwon, Hansol Im, Seong Yeol Choi, Ajay K. Monnappa, Robert J. Mitchell

**Affiliations:** School of Life Sciences, Ulsan National Institute of Science and Technology (UNIST), Eonyang-eup, Ulsan, South Korea; Indiana University Bloomington

**Keywords:** *Bdellovibrio bacteriovorus* HD100, *Chromobacterium piscinae*, cyanide, predation, violacein

## Abstract

Predation of *Chromobacterium piscinae* by *Bdellovibrio bacteriovorus* HD100 was inhibited in dilute nutrient broth (DNB) but not in HEPES. Experiments showed that the effector responsible was present in the medium, as cell-free supernatants retained the ability to inhibit predation, and that the effector was not toxic to *B. bacteriovorus*. Violacein, a bisindole secondary metabolite produced by *C. piscinae*, was not responsible. Further characterization of *C. piscinae* found that this species produces sufficient concentrations of cyanide (202 µM) when grown in DNB to inhibit the predatory activity of *B. bacteriovorus*, but that in HEPES, the cyanide concentrations were negligible (19 µM). The antagonistic role of cyanide was further confirmed, as the addition of hydroxocobalamin, which chelates cyanide, allowed predation to proceed. The activity of cyanide against *B. bacteriovorus* was found to be twofold, depending on the life cycle stage of this predator. For the attack-phase predatory cells, cyanide caused the cells to lose motility and tumble, while for intraperiplasmic predators, development and lysis of the prey cell were halted. These findings suggest that cyanogenesis in nature may be employed by the bacterial strains that produce this compound to prevent and reduce their predation by *B. bacteriovorus*.

## IMPORTANCE

Bacterial predators actively attack, kill, and enter the periplasm of susceptible Gram-negative bacteria, where they consume the prey cell components. To date, the activity of *B. bacteriovorus* HD100 has been demonstrated against more than 100 human pathogens. As such, this strain and others are being considered as potential alternatives or supplements to conventional antibiotics. However, the production of secondary metabolites by prey bacteria is known to mitigate, and even abolish, predation by bacterivorous nematodes and protists. With the exception of indole, which was shown to inhibit predation, the effects of bacterial secondary metabolites on *B. bacteriovorus* and its activities have not been considered. Consequently, we undertook this study to better understand the mechanisms that bacterial strains employ to inhibit predation by *B. bacteriovorus* HD100. We report here that cyanogenic bacterial strains can inhibit predation and show that cyanide affects both attack-phase predators and those within prey, i.e., in the bdelloplast.

## INTRODUCTION

Predation by microfaunal predators, i.e., protists and nematodes, is the chief reason for bacterial mortality in nature (1), although bacterial predators, such as *Bdellovibrio bacteriovorus* and *Bacteriovorax stolpii*, also contribute to bacterial mortality. In response to the constant threat of predation, bacteria have evolved several different mechanisms to protect themselves, including the production of secondary metabolites to combat the predator (2–4).

One well-known secondary metabolite with defensive capabilities is indole. With respect to predation, indole is both toxic to the bacterivorous nematode *Caenorhabditis elegans* (5) and capable of blocking predation of *Escherichia coli* and *Salmonella* by the bacterial predator *Bdellovibrio bacteriovorus* HD100 (6). Indole is produced by both Gram-positive and Gram-negative bacteria (7, 8) and can reach concentrations of up to 1.1 mM within mammalian guts (9, 10). With *C. elegans*, a significant loss in viability was seen when indole was present at 0.5 mM or higher (5), while concentrations between 1 and 2 mM inhibited *B. bacteriovorus* mobility and hindered its development within the bdelloplast (6).

Another secondary metabolite with known activities against predators is violacein. This bisindole antibiotic was first described more than 70 years ago (11, 12), and it has since been shown to be produced by a variety of bacterial strains, including strains of *Janthinobacterium* (13, 14), *Collimonas* (15), *Duganella* (16, 17), and *Chromobacterium* (18, 19), including *Chromobacterium piscinae* (17). Although recent studies have emphasized the activity of violacein against antibiotic-resistant strains of *Staphylococcus aureus* (17, 20, 21), this compound is also active against various protists (3, 22), and it is known to be toxic to *C. elegans* (23, 24).

In addition to violacein, *Chromobacterium violaceum* also produces cyanide (25, 26), a potent inhibitor of cytochrome *c* oxidase (27). As a secondary metabolite, cyanide is generated by *C. violaceum* using glycine through an oxidative decarboxylation step (25, 26, 28), although other amino acids may also be used (29). Studies with *Pseudomonas aeruginosa* PAO1, which is also cyanogenic, found that the cyanide produced by this bacterium was sufficient to completely kill *C. elegans* cultures (30), illustrating its potential as a deterrent against predation.

Similarly, *Serratia marcescens* is known to produce several detergents, or serrawettins, that repel *C. elegans* (31). Pradel et al*.* (31) found that purified serrawettin W2 elicited a lawn avoidance response from *C. elegans* with *E. coli* OP50, i.e., the microbe typically used to cultivate this nematode, and that this response was mediated by its sole Toll-like receptor, encoded by *tol-1*. Prior to their study (31), the role of this receptor specifically in *C. elegans* avoidance of *S. marcescens* was known (32), but how *C. elegans* recognized this pathogen was not known. Burlinson et al. (33) also describes the characterization of the *Pseudomonas fluorescens* NZ17 EDB gene cluster, whose product repels *C. elegans* but does not kill it. Although the exact compound has yet to be identified, they were able to show that it is either poorly diffusible or associated with the bacterial cell, as *C. elegans* needed to encounter *P. fluorescens* NZ17 before the aversion response was initiated (33). Furthermore, using two *C. elegans* mutants, *tax-2* and *tax-4* mutants, they also demonstrated that this response involves both chemotactic and nonchemotactic elements.

In this study, we show that *C. piscinae* is preyed upon by *B. bacteriovorus* HD100 when provided in HEPES buffer but that it was resistant when the tests were performed in dilute nutrient broth (DNB). Through a series of experiments, we identified that the effector was present within the cell-free supernatants and, similar to the EDB cluster product, it was inhibitory but not overtly toxic toward *B. bacteriovorus* HD100.

## RESULTS

### *C. piscinae* produces an inhibitor of bacterial predation.

**FIG 1:**
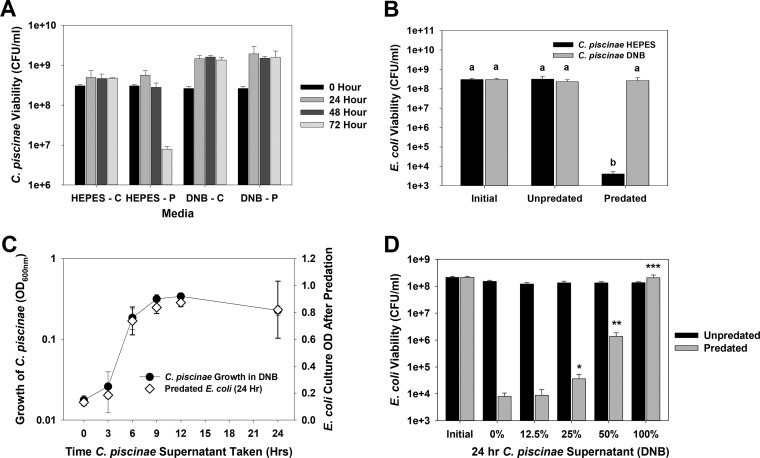
(A) Impact of the media on predation of *C. piscinae* by *B. bacteriovorus* HD100. Control cultures (unpredated) and predated cultures are indicated by the letters C and P, respectively, after the medium. A predator-to-prey ratio (PPR) of approximately 0.03 was used. Values are means plus standard deviations (error bars). This experiment was repeated eight times. (B) Filter-sterilized supernatants from *C. piscinae* grown in DNB protect *E. coli* MG1655 from predation by *B. bacteriovorus* HD100. Supernatants from *C. piscinae* cultures incubated for 24 h in HEPES or DNB were tested. The initial *E. coli* viability and unpredated and predated *E*. *coli* viability are shown. A PPR of approximately 0.03 was used. Values that are not statistically significantly different (*P* < 0.05) are indicated by the same letter (letter a or b). This experiment was repeated three times. (C) Correlation between the *C. piscinae* growth in DNB and the inhibitory activity with *E. coli* as the prey. At each time point, a sample of the *C. piscinae* culture was taken to measure the OD_600_. The cells within another aliquot were removed by filtration, and the inhibitory activity of the supernatant was assessed in predation tests with *E. coli* MG1655 as the prey. The results show a good correlation between the growth of *C. piscinae* and the inhibitory activity of its supernatant. A PPR of approximately 0.02 was used. This experiment was repeated three times. (D) The inhibitory activity of the DNB supernatants is dose dependent. Cultures of *C. piscinae* grown in DNB for 24 h were filter sterilized. The supernatants were then diluted into HEPES, and their inhibitory activity was studied using *E. coli* MG1655 as the prey. The *E. coli* viabilities were measured after 24 h. The values for pairs of samples (predated and unpredated samples) were compared. Values that are significantly different are indicated by asterisks as follows: *, *P* < 0.05; **, *P* < 0.01; ***, *P* < 0.001. This experiment was repeated four times.

*B. bacteriovorus* HD100 preyed upon *C. piscinae* in HEPES buffer but not in dilute nutrient broth (DNB) (Fig. 1A). This result suggested that *C. piscinae* produced an inhibitory molecule when provided nutrients in the form of DNB and that it may be secreted into the supernatant. This was confirmed using *E. coli* MG1655 as the prey as shown in Fig. 1B, where filter-sterilized supernatants from *C. piscinae* cultures incubated in DNB inhibited predation (Fig. 1B). Once more, parallel tests performed in HEPES found no inhibitory activity. To study this further, *C. piscinae* was grown in DNB medium (initial optical density at 600 nm [OD_600_] of 0.03), and the inhibitory activity at different time points was measured as described above with *E. coli* MG1655 as the prey (Fig. 1C). There was no apparent inhibition during the first 3 h. From 6 h on, however, predation was significantly inhibited, showing that the concentration of the inhibitor from this time point was sufficient to block *B. bacteriovorus* HD100. Using the supernatants after 24 h, we also found that the activity is dose dependent (Fig. 1D).

### Violacein is not the inhibitor.

**FIG 2:**
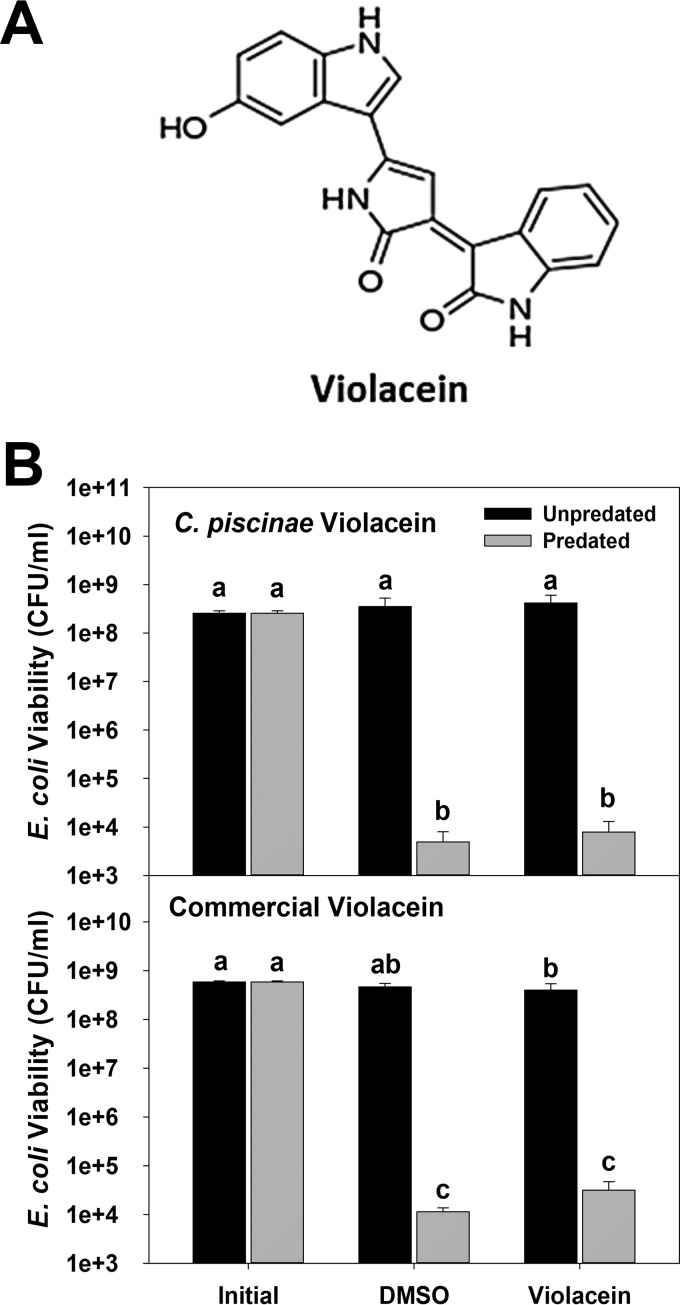
(A) Structure of violacein, a known inhibitor of microfaunal predators, i.e., nematodes and protists. This compound is produced by various bacterial strains. (B) Violacein does not negatively impact predation. Both a preparation purified from cultures of *C. piscinae* and a commercially available violacein were tested. *E. coli* MG1655 was used as the prey for *B. bacteriovorus* HD100. The initial *E. coli* viability (Initial), viability of *E. coli* cultures exposed to DMSO for 24 h with or without predation (DMSO), and viability of *E. coli* cultures exposed to 20 mg/liter violacein (in DMSO) for 24 h with or without predation (Violacein) are shown. A PPR of approximately 0.03 was used. Values are means plus standard deviations (error bars). Values that are not statistically significantly different (*P* < 0.05) are indicated by the same letter (letter a, b, or c). This experiment was repeated three times.

As one of the secondary metabolites produced by *C. piscinae* is the bisindole violacein (Fig. 2A), we initially thought that it was the inhibitor due to its structural similarity with indole (6). Cultures of *C. piscinae* grown in DNB produced, on average, between 0.3 and 0.5 mg/liter of violacein, or around 1 to 1.5 µM, although its concentration in the cell-free supernatants was close to zero, since it is hydrophobic and primarily associates with the *C. piscinae* cells. When *C. piscinae* is grown in NB medium, the violacein concentration can reach roughly 5 mg/liter (15 µM) (17). To evaluate whether violacein inhibited predation, we used the higher concentration (Fig. 2B). The same set of experiments was also performed using a commercial preparation of violacein that was produced by *Janthinobacterium lividum* to determine whether there are any differences based upon the host strain (Fig. 2B). In both cases, predation of *E. coli* MG1655 by *B. bacteriovorus* HD100 was not blocked.

### *C. piscinae* produces cyanide, which inhibits predation.

**FIG 3:**
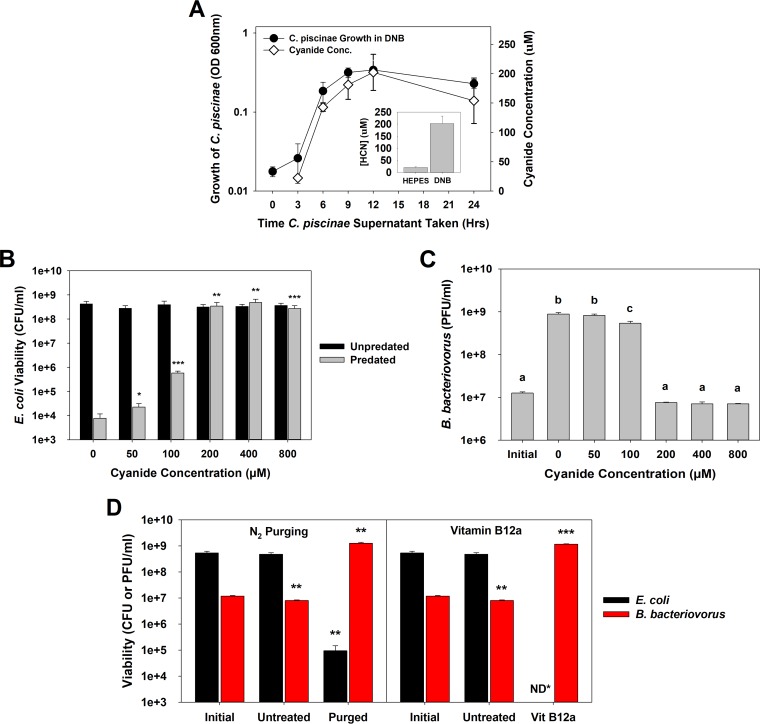
(A) Cyanogenesis by cultures of *C. piscinae* grown in DNB. The *C. piscinae* growth results are the same as in Fig. 1C. This experiment was repeated three times. (Inset) Average cyanide concentrations seen after 24 h from 13 independent experiments. (B) Potassium cyanide inhibition of predation. *E. coli* MG1655 was used as the prey strain. The surviving prey populations were determined after 24 h. The results show that cyanide concentrations of 50 µM and higher significantly inhibit predation. Statistical analyses were performed against the no-cyanide control samples. A PPR of approximately 0.03 was used. The values for pairs of values (predated and unpredated samples) were compared. Values that are significantly different are indicated by asterisks as follows: *, *P* < 0.05; **, *P* < 0.01; ***, *P* < 0.001. The experiment was performed four times. (C) *B. bacteriovorus* HD100 numbers after predation in the presence of cyanide. The population was determined using the cultures after 24 h from panel B, confirming that cyanide concentrations of 100 µM and higher significantly inhibit predation. Values that are not statistically significantly different (*P* < 0.05) are indicated by the same letter (letter a, b, or c). This experiment was repeated four times. (D) Purging of the supernatant (*n* = 3) or treating it with hydroxocobalamin (*n* = 4) restores the ability of *B. bacteriovorus* HD100 to attack. *E. coli* MG1655 was used as the prey. The medium was treated for 1 h prior to the predation experiment was performed. The values were compared to the values for the untreated control samples. The viabilities of both the predator and prey were determined after 24 h. The values were compared to the value for the respective initial populations. Values that are significantly different are indicated by asterisks as follows: **, *P* < 0.01; ***, *P* < 0.001. ND*, not detected (<10^3^ CFU/ml).

In addition to violacein, *C. piscinae* was also found to produce a significant amount of cyanide when grown in DNB medium, with an average of 202 µM after 24 h (Fig. 3A, inset). Parallel experiments within NB medium found that after 24 h, the cyanide concentration was between 600 and 800 µM, while cultures incubated in HEPES produced very little or no cyanide (Fig. 3A, inset). A strong correlation is evident when the inhibitory activities of the supernatants (Fig. 1C) are compared with their respective cyanide concentrations (Fig. 3A). In *P. aeruginosa*, the *cioAB* genes encode a cyanide-insensitive oxidase, making this organism resistant to its own cyanide production (34). A BLASTP analysis using the *P. aeruginosa* CioA and CioB amino acid sequences (NCBI GenBank accession no. CAA71555.1 and CAA71556.1, respectively) found homologues encoded by *C. piscinae* genes (*C. piscinae* CioA [NCBI accession no. KIA81027] with 48% identity with *P. aeruginosa* CioA and *C. piscinae* CioB [NCBI accession no. KIA81026] with 43% identity with *P. aeruginosa* CioB). However, neither of these genes were found within the *B. bacteriovorus* HD100 genome, helping to explain why *C. piscinae* is resistant to cyanide while this predatory bacterium is not.

The impact of cyanide on predation was further confirmed in tests using potassium cyanide (KCN) (Fig. 3B and C). When the KCN concentration was 200 µM or greater, predation was completely blocked. As shown in Fig. 3B and C, under these conditions, the prey and predator populations after 24 h were similar with their initial values, i.e., 3.1 × 10^8^ CFU/ml for *E. coli* and 1.3 × 10^7^ PFU/ml for *B. bacteriovorus* HD100. Treatment of *B. bacteriovorus* HD100 with 200 µM KCN significantly reduced the motility of this predatory bacterium. Movie S1 in the supplemental material shows the predatory cells when present in HEPES buffer alone with an average speed of 18.2 ± 4.4 µm/s (*n* = 210), while in Movie S2, they were exposed to 200 µM KCN and had an average swimming speed of only 4.5 ± 2.8 µm/s (*n* = 210). The reduced motility (6, 35) helps to explain the weaker predatory activities seen with cyanide.

10.1128/mBio.01370-17.1 MOVIE S1  Video showing *B. bacteriovorus* HD100 swimming in HEPES buffer without the addition of cyanide. Download MOVIE S1, AVI file, 2.8 MB. Copyright © 2017 Mun et al. 2017 Mun et al. This content is distributed under the terms of the Creative Commons Attribution 4.0 International license.

10.1128/mBio.01370-17.2 MOVIE S2  Video showing *B. bacteriovorus* HD100 swimming in HEPES buffer containing 200 µM cyanide. Download MOVIE S2, AVI file, 2.7 MB. Copyright © 2017 Mun et al. 2017 Mun et al. This content is distributed under the terms of the Creative Commons Attribution 4.0 International license.

To test whether cyanide alone was responsible or whether some other compound also acted as an inhibitor, two additional experiments were performed (Fig. 3D): purging and treatment of the supernatant with vitamin B_12a_. In a neutral (pH 7) environment, cyanide is present primarily as hydrogen cyanide (HCN), which is less dense than air and volatile. Taking advantage of this characteristic, we purged the media with nitrogen gas and reduced the cyanide concentration in the media from 230 µM to only 20 µM. Likewise, vitamin B_12a_, or hydroxocobalamin, acts as a scavenger of cyanide, forming cyanocobalamin (36, 37), which prevents cyanide from binding to cytochrome *c* oxidase. After either treatment, *B. bacteriovorus* HD100 was able to prey upon *E. coli* MG1655 (Fig. 3D), confirming that cyanide is the main, if not sole, inhibitor responsible.

### Cyanide halts *B. bacteriovorus* HD100 development in the bdelloplast.

**FIG 4:**
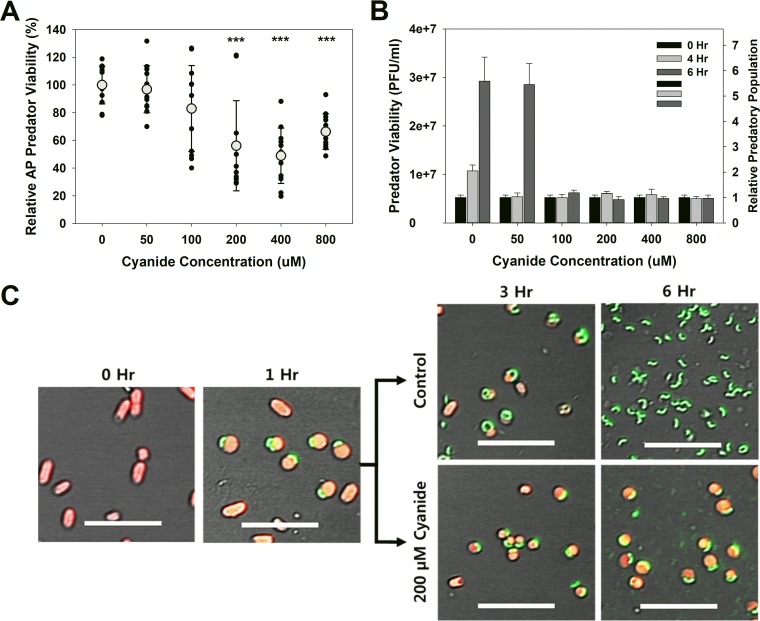
(A) Cyanide is mildly, yet significantly, toxic to attack-phase *B. bacteriovorus* HD100. *B. bacteriovorus* cultures were exposed to different micromolar concentrations of cyanide for 24 h, after which the viable populations were determined. A total of 12 independent cultures were tested. Each symbol represents the value for one culture, and the average result for a particular cyanide concentration is indicated by a light gray circle. Values were compared to the value for the no-cyanide control samples. Values that are significantly different (*P* < 0.001) are indicated (***). This experiment was repeated 12 times. (B) Cyanide prevents release of the predatory cells from the bdelloplast. This graph shows the numbers of viable predators, which include both free-swimming predators and those within the bdelloplast, seen during and after a single predation event. The relative number illustrates the titer burst seen when lysis of the bdelloplast occurs. This experiment was repeated six times. (C) Confocal microscopic images showing the impact of cyanide on the development of the predatory cells within the bdelloplasts. *E. coli* MG1655 prey express the red fluorescent protein (red), while the predator is expressing the Venus yellow fluorescent protein (green). In the presence of 200 µM cyanide, elongation and development of the intraperiplasmic predatory cells, and the resulting lysis of the bdelloplast, are halted. This agrees with the results shown in panel B. The 0-h image was taken just prior to introducing the predatory strain. Bars = 10 µm.

Although cyanide inhibited predation, it was not very toxic to attack-phase (AP) *B. bacteriovorus* HD100, as shown in Fig. 4A. The viability decreased slightly from 100 µM cyanide, with the greatest loss in viability seen at 400 µM cyanide. Even at this concentration, however, the loss in viability was only 51%. When similar experiments were performed using bdelloplasts, no toxic effect was seen for any of the concentrations tested (Fig. 4B). In the control samples, the number of predators increased after 4 h and was on average 5.6-fold higher than the initial populations. A similar increase was seen in the 50 µM samples, although there appears to have been a delay since the number of *B. bacteriovorus* did not increase until 6 h. For the higher concentrations, there was no observable increase in the predator numbers after either 4 or 6 h. When bdelloplasts within the control cultures (0 µM cyanide) and cultures treated with 200 µM cyanide were imaged using confocal microscopy, we found that the development of the intraperiplasmic predator was halted after the addition of cyanide (Fig. 4C). In the control cultures, the predators within the bdelloplasts were elongated at 3 h, while only AP *B. bacteriovorus* HD100 cells were seen at 6 h, since lysis of the prey had already occurred. In contrast, the addition of 200 µM cyanide after 1 h halted the growth and development of the intraperiplasmic predator, as shown in Fig. 4C. Even after 6 h, the bdelloplasts are essentially indistinguishable from those imaged just before cyanide was added.

## DISCUSSION

In this study, we show that predation of *C. piscinae* by *B. bacteriovorus* HD100 occurs in HEPES buffer, although it took 3 days to see a 60-fold drop in prey viability. Compared to other prey strains, this is rather protracted and implied that *C. piscinae* is inherently resistant to predation by *B. bacteriovorus* HD100. When the same tests were performed in DNB, there was no loss in the viability of *C. piscinae*, a result that implied that this bacterium produces an inhibitor when provided with amino acids. The only other report found describing the predation of a *Chromobacterium* strain was a study by Jurkevitch et al. (38). They found that four out of the five predatory strains they tested were capable of predating on *C. violaceum* but used HEPES buffer as the medium. On the basis of our findings here, this would be permissive for predation to occur.

Violacein is formed by a variety of bacterial strains, including *C. piscinae* (17), through a condensation reaction involving two tryptophan molecules (39, 40). Given violacein’s bisindole chemical structure and the established activity of indole against *B. bacteriovorus* HD100 (6), we initially thought that this compound was the *C. piscinae* inhibitor being produced. According to Choi et al. (17), *C. piscinae* produces slightly less than 4.5 mg/liter of violacein when grown for 24 h in NB medium. Although this is equal to a concentration of 15.4 µM, which is far lower than the 1 mM needed for indole to inhibit predation (6), it was much higher than what was actually seen in the DNB cultures (1.2 µM). Tests with both crude and commercial violacein preparations at the higher concentration found that this compound does not inhibit predation, consequently, violacein is not responsible for the loss in activity seen.

In addition to violacein, *C. piscinae* also produced cyanide when grown in DNB medium. Although the production of cyanide by *C. violaceum* was established long ago (25, 41, 42), this is the first report demonstrating that *C. piscinae* also produces cyanide. Cyanide is formed during the oxidative decarboxylation of glycine (25, 26, 28) and of glutamate and methionine (29). As the DNB medium used in this study contains both beef extract and an enzymatic digest of gelatin, all three of these amino acids are provided in this medium. When grown in DNB, *C. piscinae* produced an average of 202 µM cyanide after 24 h, although most (143 µM) was produced within the first 6 h (Fig. 3A). The production trend in Fig. 3A is quite similar with that published by Michaels et al. (25) during their characterization of *C. violaceum*. In both cases, there was a lag period that lasted for the first 4 h, after which the cyanide concentrations rapidly increased. The maximum cyanide concentration obtained with *C. violaceum* was approximately 16 mg/liter, or 600 µM, which is threefold higher than that produced by *C. piscinae*, i.e., 202 µM. Aside from likely variations in the two strains, this difference can be attributed to the composition of the medium used in each study, i.e., DNB (0.8 g/liter) here and 10 g/liter peptone in their study, as Michaels et al. (25) also reported that peptone greatly influenced the final cyanide yields with *C. violaceum*. As a demonstration of this, when *C. piscinae* was grown in NB (8 g/liter), the final cyanide concentrations were comparable at 600 to 800 µM.

Aside from *C. violaceum* and *C. piscinae*, bacterial cyanogenesis is best characterized in pseudomonads (28, 43), where it has been linked to both reduced nematode viability and egg hatching rates (30, 33, 44, 45). For instance, exposure to *P. aeruginosa* PAO1 completely (100%) killed *C. elegans* cultures, while only 15% of the nematode population was killed by an isogenic noncyanogenic mutant strain (30). Likewise, in Siddiqui et al. (44), the egg hatching rates for *Meloidogyne javanica* in control cultures were 90%. This rate dropped significantly (to 40%) when the cultures were exposed to the cyanogenic *Pseudomonas protegens* CHA0, but it dropped only slightly (85%) with *P. protegens* CHA77, an isogenic mutant lacking the ability to produce cyanide. Both studies illustrate the activity of cyanogenic bacteria against predatory nematodes and the potential benefit they gain from producing cyanide.

To date, bacterial cyanogenesis has been identified only in Gram-negative microbes (46), and this may be evolutionarily important given the selective activity of *B. bacteriovorus*, which attacks only Gram-negative strains. Cyanide acts by binding to and inhibiting cytochrome *c* oxidase (27), a key enzyme in the respiratory electron transport chain, making it a potentially strong poison for *B. bacteriovorus* HD100, since this microbe is regarded as a strict aerobe.

Although the effect cyanide has on bacterial predation has not been reported, it was studied from the perspective of electron transport systems of *B. bacteriovorus* and oxidation of NADH using attack-phase predatory cells or cell lysates (47). The authors found that 5 mM KCN completely inhibited respiration, while 0.5 mM KCN reduced NADH oxidation by 75% in cell-free extracts of *B. bacteriovorus* strain 6-5-S. Similarly, another study reported that the amino acid uptake by *B. bacteriovorus* 109D (H-I), a host-independent variant that can grow axenically, was inhibited by as much as 91% by 500 µM cyanide, while lower concentrations had lesser impact (48). Moreover, transport of phosphate into both *B. bacteriovorus* 109J and 109D was completely inhibited by 1 mM cyanide.

Although both studies used cyanide concentrations that were higher than the inhibitory concentrations found in this study, i.e., 100 to 200 µM, their findings help to explain the activity of cyanide as seen here. As cyanide impairs electron transport and energy production, as well as inhibits transport of critical nutrients (i.e., phosphate and amino acids) needed for the growth and development of the predator within the bdelloplast, the implication would be a halting of growth for intraperiplasmic predators when the cyanide concentration is sufficiently high. This is what was observed in Fig. 4C. However, our findings also clearly demonstrate that the effects of cyanide were not permanent, since exposed *B. bacteriovorus* cells still formed plaques and grew later when the cyanide was diluted. This was true for both the free-swimming *B. bacteriovorus* (Fig. 4A) as well as those within bdelloplasts (Fig. 4B). This is further exemplified in Movie S2 in the supplemental material, where predatory cells exposed to cyanide were still viable, although their ability to swim was severely impaired. This impairment was anticipated, since the flagellar motor activity is dependent upon the proton motive force, which is generated by the electron transport chain (ETC). Given that cyanide binds to cytochrome *c* oxidase, a member of the ETC, and inhibits any further electron transport, the proton motive force would quickly be spent and bacterial motility partially halted. This was seen in both *P. fluorescens* (49) and *E. coli* (50). For *P. fluorescens*, 2 min was sufficient to achieve a stable level of inhibition, illustrating the rapid loss of motility during exposure. For *E. coli*, exposure to 100 µM cyanide led to both a 63% reduction in the average speed of this bacterium and a 38% reduction in the number of cells that were motile. Both of these studies illustrate the same response as seen with *B. bacteriovorus* HD100 when exposed to cyanide in this study.

### Conclusions.

In addition to the clear impact bacterial cyanogenesis has on macrofaunal predators (i.e., nematodes and protozoa), this study illustrates an additional benefit for cyanogenic bacterial strains, namely, that they may be shielded and protected from bacterial predation. The amount of cyanide produced by *C. piscinae* when provided DNB was sufficient to inhibit predation by *B. bacteriovorus* and halt the development of the predator within the bdelloplast. From an ecological perspective, this suggests that bacterial cyanogenesis may be used to protect natural microbial communities from predation by *B. bacteriovorus*. Given the small amount of nutrients present in this medium and the clear impact they had on the predation results, this study also suggests that caution should be taken in defining bacterial strains that are susceptible to predation and those that are resistant, as secondary metabolites clearly can play a role.

## MATERIALS AND METHODS

### Strains and culturing methods.

The strains used in this study were *Bdellovibrio bacteriovorus* HD100 (DSMZ 50701), *Chromobacterium piscinae* LMG 3947, and *E. coli* MG1655 (ATCC 700926). Culturing of *B. bacteriovorus* HD100 was performed as described previously (51, 52) using *E. coli* MG1655 as the prey. *C. piscinae* and *E. coli* MG1655 were grown from frozen stocks on nutrient broth (NB) agar plates at 30°C overnight. A single colony was inoculated into fresh NB medium and grown for 24 h at 30°C and 250 rpm.

For the predation studies, the prey cells were pelleted by centrifugation (15 min, 2,000 × *g*) and resuspended to an optical density at 600 nm (OD_600_) of 1.0 in either dilute nutrient broth (DNB; 1/10 NB) or HEPES buffer (pH 7.2), both supplemented with 3 mM MgCl_2_ and 2 mM CaCl_2_. The cultures were split in half, and one half had *B. bacteriovorus* HD100 added to a predator-to-prey ratio (PPR) of approximately 0.03. The other half was used as an unpredated control sample. The initial prey and predator viabilities and viabilities after 24 h were determined as described previously using top agar plates (51).

### *C. piscinae* supernatant experiments.

*C. piscinae* was cultured as described above for 24 h in NB (30°C and 250 rpm), after which the culture was diluted 1:100 into either HEPES buffer or DNB medium. These cultures were incubated for an additional 24 h at 30°C and 250 rpm. At this time, the supernatants were collected by removing the *C. piscinae* cells through centrifugation (15 min, 2,000 × *g*) and sterilized using a 0.22-µm filter (Millipore, USA). To these supernatants (DNB and HEPES), *E. coli* MG1655 was added as described above to an OD_600_ of 1.0. The cultures were split in half, and one half had *B. bacteriovorus* HD100 added to a predator-to-prey ratio (PPR) of approximately 0.03. The other half was used as an unpredated control sample. The initial prey and predator viabilities and the viabilities after 24 h were determined as described previously (51). A similar protocol was used for the supernatant dose-dependent experiments. For these tests, the cell-free *C. piscinae* DNB supernatants were diluted using HEPES buffer to achieve the desired percent supernatant. *E. coli* MG1655 was then added to an OD_600_ of 1.0, and the predation tests were performed as described above.

To study the impact of the *C. piscinae* growth stage on predation, DNB cultures were prepared as described above, and samples were taken at set times (0, 3, 6, 9, 12, and 24 h) for OD_600_ measurement and predation tests. The *C. piscinae* cells were removed from these samples as described above using centrifugation and filtration. *E. coli* MG1655 was resuspended in the supernatant samples to an OD_600_ of 1.0, and *B. bacteriovorus* HD100 was added to a PPR of approximately 0.03. After 24 h at 30°C and 250 rpm, the OD of the different cultures were measured at 600 nm.

### Violacein preparation and tests.

To test the effects of violacein, we purchased violacein from Sigma-Aldrich (USA), prepared using *Janthinobacterium lividum*, as well as purified violacein from the *C. piscinae* cultures grown in our lab. To purify violacein, we used the ethanol extraction method as described previously (17) followed by crystallization using acetone (11). The concentration of violacein within the samples was determined using high-performance liquid chromatography (HPLC) as described previously (17). This method was used for both the preparation and the supernatant samples.

Stock solutions (100×) containing 0.05 g/liter and 0.5 g/liter of either violacein preparation were prepared in dimethyl sulfoxide (DMSO) (Sigma-Aldrich, USA). To *E. coli* MG1655 cultures in HEPES, prepared as described above to an OD_600_ of 1.0, the violacein stock was added at a 1:100 dilution. The cultures were split in half, and immediately afterwards, *B. bacteriovorus* HD100 was added to one of the tubes to give a PPR of approximately 0.03. All the tubes were incubated at 30°C with shaking at 250 rpm for 24 h. The prey and predator viabilities were determined initially (0 h) and after 24 h.

### Cyanide concentration determination.

To measure the cyanide concentrations in the culture media, we used a modified method based upon previous studies (53, 54). Briefly, 0.1 M *o*-dinitrobenzene (Fluka, Germany) and 0.2 M *p*-nitrobenzaldehyde (Sigma-Aldrich, USA) solutions were prepared in 2-methoxyethanol (Sigma-Aldrich, USA). For each measurement, a fresh 1:1 mixture of these two solutions was made, and this was mixed 77:23 with the supernatant samples (100 µl total). Afterwards, 1.8 µl of 5 M NaOH was added to each sample, giving a final concentration of 0.09 M NaOH, which was required to adjust the pH prior to measurement. The supernatants were diluted as needed to ensure that the cyanide concentration was within the measurable range. After mixing, the samples were incubated for 30 min at room temperature, and then 900 μl of 2-methoxyethanol was added to each tube. From each sample, 100 µl was aliquoted into the wells of transparent 96-well plates (SPL, South Korea), and the OD_578_ was determined. The cyanide concentrations were calculated using a calibration curve prepared with known KCN solutions.

### Purging and hydroxocobalamin experiments.

The *C. piscinae* cell-free DNB supernatants were prepared as described above. Each supernatant sample (20 ml) was then purged with nitrogen gas (flow rate, 3 liters/min) for 1 h. After purging, the medium was reconstituted to 20 ml using sterile water, and the predation experiments were performed as described above using *E. coli* MG1655 as the prey.

Hydroxocobalamin (vitamin B_12a_) was purchased from Sigma-Aldrich (USA). Stock solutions of this vitamin were prepared using sterile water and subsequently filter sterilized before use. For the experiments, the *C. piscinae* cell-free DNB supernatants were prepared as described above. To each, 200 µM hydroxocobalamin was added just prior to introducing the prey or predator.

### Potassium cyanide inhibition of predation.

Potassium cyanide (KCN) was purchased from Sigma-Aldrich (USA). A stock solution (1 M) was prepared using sterile deionized water and filter sterilized (0.22-µm syringe filter; Millipore [USA]). The cyanide stock was diluted to a 2× concentration using sterile HEPES buffer and subsequently mixed 1:1 (vol/vol) with an *E. coli* culture, prepared in HEPES to an OD of 2.0. Immediately afterwards, *B. bacteriovorus* HD100 was added to the samples to obtain a PPR of approximately 0.03. After 24 h at 30°C and 250 rpm, the surviving *E. coli* and resulting *B. bacteriovorus* HD100 populations were determined.

### Potassium cyanide impact on attack-phase and intraperiplasmic predator viabilities.

The KCN stock solution was diluted into cultures of *B. bacteriovorus* HD100 to achieve the desired concentration. These cultures were then incubated at 30°C and 250 rpm for 24 h, after which the number of surviving *B. bacteriovorus* HD100 was determined. For the intraperiplasmic studies, predation of *E. coli* was performed for 1 h at 30°C and 250 rpm using a PPR of approximately 0.03. At this time, KCN was added to achieve the desired concentration. The number of predators were determined after an additional 3 and 5 h using top agar plates as described previously (52).

### Confocal microscopy.

For the microscopic images, we used *B. bacteriovorus* HD100 bearing plasmid pMQ572, which expresses the Venus yellow fluorescent protein (55), and *E. coli* expressing the Dsred fluorescent protein via plasmid pHTK3 (56). The pMQ572 plasmid for *B. bacteriovorus* HD100 was constructed by Robert Shanks’ group and is similar to another construct that expresses the fluorescent tdTomato protein (31, 57). pMQ572 was introduced into *B. bacteriovorus* HD100 by conjugation as described previously (58) with slight modifications. For these experiments, approximately 2 × 10^10^ *B. bacteriovorus* HD100 bacteria were pelleted by centrifugation (16,000 × *g*, 15 min) and resuspended in 2 ml HEPES. The donor strain, *E. coli* S17λpir/pMQ572, was grown to stationary phase (16 h) in LB medium with 10 µg/ml gentamicin added at 30°C and 250 rpm. The cells were then pelleted by centrifugation (20 ml, 16,000 × *g*, 15 min) and resuspended in 2 ml HEPES buffer. Both the donor and recipient (50 μl) were mixed and spotted onto nitrocellulose filters (0.22-μm pore size) (Pall Life Sciences, USA), which were placed on top of DNB agar plates. The plates were then incubated overnight at 30°C. After 24 h, the bacteria were resuspended in 2 ml HEPES buffer and serially diluted. These samples were used to prepare DNB top agar plates with *E. coli* MG1655 harboring pMQ572 as the prey. These plates had 10 μg/ml gentamicin added to them to select for the transconjugants. The plates were incubated at 30°C until plaques were visible. The plaques were then excised, and the recombinant predatory cells were grown in HEPES buffer with gentamicin added using *E. coli* MG1655/pMQ572 as the prey. It was noticed that when these predatory cell cultures were grown in DNB, they were more fluorescent than cultures in HEPES, and thus, DNB was used to prepare the recombinant cultures for the experiments. Consequently, the predation tests were conducted as described above in DNB except the PPR was approximately 1.0. After 1 h, the culture was divided, and KCN was added to a final concentration of 200 µM. Samples were taken at set times and imaged using a Carl Zeiss LSM 780 multiphoton confocal microscope controlled by ZEN 2012 software.

### Statistical analyses.

All experiments were performed at least three times. Statistical analysis was done using Student’s *t* test to compare two sets of data). For comparing three or more data sets, analysis of variance (ANOVA) was performed followed by the Tukey posthoc test.

### Data availability.

The bacterial strains used in this study are available at various strain repositories (LMG [Belgian Coordinated Collection of Microorganisms], DSM [Deutsche Sammlung von Mikroorganismen und Zellkulturen GmbH], and ATCC [American Type and Culture Collection]).
